# Expressing the Human Cholesteryl Ester Transfer Protein Minigene Improves Diet-Induced Fatty Liver and Insulin Resistance in Female Mice

**DOI:** 10.3389/fphys.2021.799096

**Published:** 2022-01-10

**Authors:** Lin Zhu, Julia An, Sivaprakasam Chinnarasu, Thao Luu, Yasminye D. Pettway, Kelly Fahey, Bridget Litts, Hye-Young H. Kim, Charles R. Flynn, MacRae F. Linton, John M. Stafford

**Affiliations:** ^1^VA Tennessee Valley Healthcare System, Nashville, TN, United States; ^2^Division of Diabetes, Endocrinology, and Metabolism, Vanderbilt University School of Medicine, Nashville, TN, United States; ^3^Department of Chemistry, Vanderbilt University, Nashville, TN, United States; ^4^Section of Surgical Sciences, Vanderbilt University School of Medicine, Nashville, TN, United States; ^5^Atherosclerosis Research Unit, Department of Medicine, Vanderbilt University School of Medicine, Nashville, TN, United States; ^6^Department of Molecular Physiology and Biophysics, Vanderbilt University, Nashville, TN, United States

**Keywords:** CETP in females, PPARalpha, polyunsaturated fatty acid (PUFA), fatty acid oxidation, insulin resistance, LXR, fatty liver

## Abstract

Mounting evidence has shown that CETP has important physiological roles in adapting to chronic nutrient excess, specifically, to protect against diet-induced insulin resistance. However, the underlying mechanisms for the protective roles of CETP in metabolism are not yet clear. Mice naturally lack CETP expression. We used transgenic mice with a human CETP minigene (huCETP) controlled by its natural flanking region to further understand CETP-related physiology in response to obesity. Female huCETP mice and their wild-type littermates were fed a high-fat diet for 6 months. Blood lipid profile and liver lipid metabolism were studied. Insulin sensitivity was analyzed with euglycemic-hyperinsulinemic clamp studies combined with ^3^H-glucose tracer techniques. While high-fat diet feeding induced obesity for huCETP mice and their wild-type littermates lacking CETP expression, insulin sensitivity was higher for female huCETP mice than for their wild-type littermates. There was no difference in insulin sensitivity for male huCETP mice vs. littermates. The increased insulin sensitivity in females was largely caused by the better insulin-mediated suppression of hepatic glucose production. In huCETP females, CETP in the circulation decreased HDL-cholesterol content and increased liver cholesterol uptake and liver cholesterol and oxysterol contents, which was associated with the upregulation of LXR target genes in long-chain polyunsaturated fatty acid biosynthesis and PPARα target genes in fatty acid β-oxidation in the liver. The upregulated fatty acid β-oxidation may account for the improved fatty liver and liver insulin action in female huCETP mice. This study provides further evidence that CETP has beneficial physiological roles in the metabolic adaptation to nutrient excess by promoting liver fatty acid oxidation and hepatic insulin sensitivity, particularly for females.

## Introduction

In humans, cholesteryl ester transfer protein (CETP) shuttles cholesteryl esters from HDL to apoB-containing particles (VLDL and LDL) and transfers triglycerides (TG) from apoB-containing particles into HDL in the circulation. CETP-mediated shuttling of cholesteryl esters from HDL to apoB-containing particles is part of the reverse cholesterol transport process where cholesterol is delivered to the liver for secretion as bile acids (Agellon et al., 1991). CETP-mediated shuttling of TGs to HDL accelerates the clearance of HDL’s scaffold protein apoA1 and decreases HDL-cholesterol levels (Lamarche et al., 1999). HDL-cholesterol levels are inversely associated with the risk of cardiovascular disease (CVD) and epidemiological studies have shown that low blood CETP activity is associated with low CVD risk in certain populations (Khera et al., 2010; Ritsch et al., 2010; Robins et al., 2013). CETP inhibition has been pursued as an approach to increase HDL-cholesterol levels and reduce CVD risk. Most clinical trials have not shown that CETP inhibition reduces CVD risk (Barter et al., 2007; Kastelein et al., 2007; Lincoff et al., 2017), although anacetrapib had modest CVD risk reduction after 4 years of follow-up (Group et al., 2017). Of note, approximately 2/3 of patients in clinical trials with CETP inhibitors were obese (Barter et al., 2007; Kastelein et al., 2007; Group et al., 2017; Lincoff et al., 2017). Clinical studies have also shown that the association between blood CETP levels and CVD risk depends on plasma TG levels and sex (Boekholdt et al., 2004; Robins et al., 2013). In the EPIC-Norfolk cohort study, plasma CETP levels were only associated with the risk of future CVD in non-obese subjects whose plasma TG levels were higher than the median (Boekholdt et al., 2004). Obesity and hyperlipidemia are known to significantly impact aspects of cholesterol metabolism and HDL biology and may impact aspects of CETP-mediated biology (Cappel et al., 2013; Palmisano et al., 2016; Zhu et al., 2018a; van den Boogert et al., 2020).

CETP transgenic mice have been used to study CETP-related biology since mice naturally lack CETP expression (Marotti et al., 1993; Cappel et al., 2013; Palmisano et al., 2017; Raposo et al., 2021). Similar to observations in human studies, the impact of transgenic CETP expression in mice with regard to atherosclerosis risk factors is not consistent. Studies show that in atherosclerotic-prone *Ldlr^–/–^* or *apoE^–/–^* mice, CETP expression increases atherosclerotic burden because of the redistribution of cholesterol in lipoprotein particles in the setting of impaired LDL clearance (Marotti et al., 1993; Plump et al., 1999; Westerterp et al., 2006). However, in *apoCIII*-transgenic hypertriglyceridemic mice or SR-BI knockout mice, CETP expression limits atherosclerosis progression, suggesting CETP overexpression could be atheroprotective in the presence of hypertriglyceridemia (Hayek et al., 1995; Forrester et al., 2005; Harder et al., 2007).

In addition to modifying HDL functionality, CETP has been shown to play a role in liver lipid metabolism and TG production and clearance in the circulation (Marotti et al., 1992; Cappel et al., 2015; Palmisano et al., 2016; Zhu et al., 2018a). We previously showed that the overexpression of simian CETP in mice protects against diet-induced insulin resistance (Cappel et al., 2013; Palmisano et al., 2016). With transgenic overexpression of the simian CETP gene driven by a mouse metallothionein (MT1) promoter, CETP promotes liver lipid oxidation and VLDL-TG production in chow-fed female mice (Marotti et al., 1992; Palmisano et al., 2016) and increases biliary cholesterol secretion and improves insulin sensitivity in HFD-fed female mice (Cappel et al., 2013). The presence of CETP in mice carrying the human CETP minigene directs cholesterol elimination away from the intestinal pathway toward the biliary pathway during high-fat high-cholesterol feeding (Li et al., 2019). Recent studies have shown a novel role of CETP to attenuate adiposity by enhancing lipolysis and brown adipose tissue activity (Raposo et al., 2021). Pharmacological inhibition of CETP in transgenic mice improves HDL function with regard to cholesterol efflux and anti-oxidative capacities but worsens the anti-inflammatory capacity of HDL and decreases insulin sensitivity during a high-fat diet (HFD) feeding (Zhu et al., 2018a). This study also suggested that, while increasing HDL-cholesterol level, inhibition of serum CETP activity leads to metabolic dysregulation such as fatty liver, insulin resistance, and inflammation (Zhu et al., 2018a). Mounting evidence has shown that CETP is directly or indirectly involved in multiple pathways of lipid metabolism. Studying the physiological roles of CETP is important to further understand the metabolic connection between CETP-related physiology and CVD risk.

To better understand CETP-related physiology in humans, we sought to further investigate the lipid metabolic pathways with mice expressing human CETP minigene (huCETP) controlled by its natural flanking sequences. In contrast to transgenic mice overexpressing the simian CETP driven by a constitutive promoter, mice expressing huCETP have blood CETP levels and a tissue CETP expression pattern similar to that seen in humans (Agellon et al., 1991; Jiang et al., 1992). With this mouse model, we found that the expression of huCETP improved hepatic and whole-body insulin sensitivity in female but not male mice during obesity. In addition, we found that the biosynthesis pathway for long-chain polyunsaturated fatty acid is up-regulated in the liver of female huCETP transgenic mice, which was associated with the increased liver fatty acid β-oxidation and reduced liver fat content, further supporting the protective role of CETP in lipid and lipoprotein metabolism with obesity.

## Materials and Methods

### Cholesteryl Ester Transfer Protein Transgenic Mice and Euglycemic-Hyperinsulinemic Clamp Study

Transgenic mice expressing the human CETP minigene (huCETP) on a C57BL/6J genetic background were purchased from the Jackson Laboratory (B6.CBA Tg(CETP) 5203Tall/J). Transgenic mice were crossed with C57BL/6J mice (strain 000664) and were housed at 23^°^C with a 12-h-light/12-h-dark cycle. Transgenic simian CETP mice (sCETP) mice were from Jackson Laboratory (C57BL/6-Tg(CETP)1Pnu/J, strain 001929) as we described before (Martinez et al., 2012; Cappel et al., 2013; Zhu et al., 2018a). Transgenic huCETP mice, wild-type (WT) littermates, and sCETP mice were genotyped with semi-quantitative PCR and confirmed by immunoblotting for blood CETP. Twelve-week-old (12 ± 1 weeks) mice were fed a high-fat diet (60% of calories were from fat, 20% of calories were from protein, Research Diets D12492) for 6 months. The carotid artery and jugular vein were catheterized at the Mouse Metabolic Phenotyping Center (MMPC) at Vanderbilt University Medical Center as previously described (Zhu et al., 2013). After 5 days, mice underwent a euglycemic-hyperinsulinemic clamp study as we reported before (Cappel et al., 2013; Zhu et al., 2013, 2018a). Hyperinsulinemia was maintained by a consistent infusion of insulin at 4 mU/kg/min and euglycemia (∼150 mg/dL) was maintained by measuring blood glucose every 10 min starting at *t* = 0 and adjusting an infusion of 50% dextrose as necessary. Mice received saline-washed erythrocytes from donors to prevent a fall in hematocrit. The clamp study was performed for 120 min, and blood samples were collected at *T* = 30 and *T* = 60 min during clamps. Mice were sacrificed at the end of the 120 min of the clamp, and blood was collected, and tissues were flash frozen. All procedures were performed following the National Institutes of Health Guidelines for the Care and Use of Animals and were approved by the Institutional Animal Care and Use Committee at Vanderbilt University.

### Blood Chemistry and Cholesteryl Ester Transfer Protein Activity

Mouse blood samples were collected after 5 h of fasting before the insulin clamp study. Fasting serum cholesterol and triglyceride levels were determined using Infinity™ Cholesterol and Triglycerides reagents from Thermo Scientific according to the manufacturer’s instructions (Cat# TR13421 and TR22421).

Blood inflammatory factors interleukin-6 (IL-6) and monocyte chemoattractant protein-1 (MCP-1) and other chemistry parameters were determined by the Vanderbilt Hormone Assay Core laboratory.

Blood insulin concentrations were determined using Rat/Mouse Insulin ELISA Kit from Millipore following the manufacture’s instruction (Cat#EZRMI-13K).

The second cohort of mice was prepared with a high-fat diet feeding for measuring blood ketone body levels. Blood ketone body levels at different time points of fasting were determined using the Precision Xtra Blood Glucose and Ketone Monitoring System (Abbott, NDC57599-8814-1).

The third cohort of mice was prepared with a high-fat diet and sacrificed after 5 h of fasting for determining liver TG content during fasting.

Blood CETP activity was measured using a cell-free assay according to the manufacturer’s protocol (Roar Biomedical) as we reported previously (Zhu et al., 2018a). Fasting blood from female huCETP mice (*n* = 6) and age-matched female simian CETP mice (*n* = 4) and female wild-type mice (*n* = 6) fed a chow diet were used for blood CETP activity assay. Human blood samples for the CETP activity assay were purchased fresh frozen plasma and de-identified samples from the Vanderbilt Lipid Clinic and Laboratory.

We used size exclusion chromatography (SEC) with a high-performance liquid chromatography (HPLC) system to separate plasma lipoproteins to quantify the distribution of cholesterol. The SEC separation was performed as described with a superose™ 6 10/300 GL from GE Healthcare (Cat# 17-5172-01) (Zhu et al., 2018b). Fractional cholesterol content was assayed using Infinity™ Cholesterol reagent (Cat# TR13421). VLDL was defined as fractions 8–16, LDL was defined as fractions 17–29, and HDL as fractions 30–6.

### Western Blotting

Western blotting for liver proteins was performed as reported previously (Zhu et al., 2013). The primary antibody for CETP (ab51771, ab157183) was from *Abcam*; antibodies for SR-BI (NB400-104) and PDK4 (NBP1-07047) were from *Novus*, antibodies for DGAT1 (sc-32861) and DGAT2 (sc-668549) were from *Santa Cruz*; antibodies for AKT (Cat# 9272), pAKT (Cat# 4060), ERK (Cat# 9107), pERK (Cat# 4370), ACC (Cat#3676), and pACC (Cat#3661) were from *Cell Signaling*. The following primary antibodies were all from *Abcam*: antibody for LDL receptor (ab30532); anti-SCD1 (ab19862); anti-PGC-1α (ab54481); antibodies for ELOVL2 (ab176327) and ELOVL5 (ab205535); antibodies for FADS1 (ab126706) and FADS2 (ab170665), antibody for PPARα (ab8934), antibody for ABCA1 (ab66217), antibody for HMGCS2 (ab137043), antibody for MCAD (ab110296), and antibody for CPT1α (ab128568). β-actin was used as loading control for liver tissues and detected with either rabbit anti-actin (I-19) antibody (sc-1616) from *Santa Cruz* or with mouse anti-human actin antibody (MCA5775GA) from Bio-Rad. α-Tubulin was used as loading control for muscle tissues and detected with an antibody from Abcam (ab4074). All primary antibodies were diluted at 1:1,000, except where otherwise noted, and incubated at 4^°^C overnight. All secondary antibodies were from *LI-COR Odyssey* (Donkey anti-mouse IRDye ^®^680 Cat#926-32222; Donkey anti-mouse IRDye ^®^800CW Cat#926-3212; Donkey anti-rabbit IRDye ^®^680 Cat#92668023; Goat anti-rabbit IRDye ^®^800CW Cat#926-32211) and diluted at 1:10,000 or 1:15,000 and incubated at room temperature for 1 h. Images were acquired using an *LI-COR Odyssey* infrared imaging system (*LI-COR Biotechnology*). Blot density was quantified and analyzed with Image J and plotted with Prism.

### Liver Lipids and mRNA Quantification

Liver lipid content including triglycerides, cholesterol, diacylglycerol, and fatty acid compositions in triglycerides and phospholipids from the 1st cohort mice were analyzed at the Vanderbilt University Medical Center Lipid Core.

Liver TG content during fasting from the 3rd cohort mice were determined with the method we reported previously (Luo et al., 2013). Briefly, liver tissue from each mouse was accurately weighted (∼50 mg/sample) and liver lipids were extracted using Folch methods. TGs were first separated by thin-layer chromatography with heptane/isopropyl ether/acetic acid (60:40:3 *v*/*v*), and then isolated and analyzed with Infinity™ Triglycerides reagents.

Total RNA was isolated from approximately 50 mg of snap-frozen tissues using a RNeasy mini kit (Qiagen). cDNA was synthesized using the Quanta qScript cDNA synthesis kit (Quantabio). Real-time PCR was performed using TaqMan™ Fast Universal PCR Master Mix (2X) and TaqMan Gene Expression assays (Life Technologies, Fads1: Mm00507605_m1; Fads2: Mm00441203_m1; 18S_ Hs99999901_s1) on the QuantStudio 12K Flex Real-Time PCR System (Applied Biosystems).

### LC-MS/MS Analysis of Liver Oxysterols

Liver samples were weighed (50–100 mg) and lysed in 0.5 ml tissue lysis buffer containing 0.9%NaCl, 50 mM HEPES, 0.01% TritonX100, 0.1% SDS, protease inhibitors and 10 μL antioxidant solution (2.5 mg TPP and 1.0 mg BHT in 1 mL EtOH). 10 μL *d*_7_-ketocholesterol (10 μM) internal standard, 400 μL 0.9% NaCl, and 600 μL Folch solution (2:1 = CHCl_3_: MeOH, v:v) were added to 100 μL of each sample. The CHCl_3_ layer was collected, dried, and derivatized by the addition of 100 μL of a fresh-made N,N-dimethylglycine (DMG) solution (Per 1 mL of reagent: 20 mg 2-Methyl-6-nitrobenzoic anhydride, 14 mg DMG 6 mg DMAP, 0.1 mL anhydrous Et_3_N in 0.9 mL anhydrous CHCl_3_). After 30 min agitation at room temperature, the resulting solution was evaporated in Speed Vacuum Concentrator and reconstituted in 100 μL of methanol (Tallman et al., 2021). Ten microliter was injected onto the column (Agilent Poroshell EC, 10 cm × 2.1 mm, 1.9 μm) using a solvent mixture of CH_3_CN: MeOH: H_2_O, 70:25:5 (0.01% (v) formic acid, 1 mM NH_4_OAc) for 10 min runtime at a flow rate of 400 μL/min. A TSQ Quantum Ultra mass spectrometer (Thermo Fisher Scientific) was used for MS detections, and data were acquired with a Finnigan Xcalibur software package. Selected reaction monitoring (SRM) of the DMG derivatives was acquired in the positive ion mode using electrospray ionization (ESI). The SRM transition of precursor to product ion included *m/z* 493.4 → 390.4 for DMG-*d*_7_-7keto-cholesterol, 573.5 → 470.5 for DMG-4βOH-cholesterol, 486.3 → 383.3 for DMG-7keto-cholesterol, 488.3 → 367.3 for DMG-25OH-cholesterol, 573.5 → 367.4 for DMG-22βOH-cholesterol, 573.3 → 470.3 DMG-27OH-cholesterol. The response factors of other oxysterols were determined against *d*_7_-7keto-cholesterol. Final sterol numbers are reported as nmol/mg tissues.

### Statistical Analysis

All measurements passed D’Agostino and Pearson omnibus normality test (alpha = 0.05). Differences between groups were determined by ANOVA followed by multiple comparison tests or by Student’s *t*-test with Welch’s correction as appropriate. Significance was flagged by *P* < 0.05. Data were presented as means ± SD unless otherwise indicated.

## Results

### Serum Cholesteryl Ester Transfer Protein Activity in Human Cholesteryl Ester Transfer Protein Minigene Mice Is Comparable to That in Humans

**FIGURE 1:**
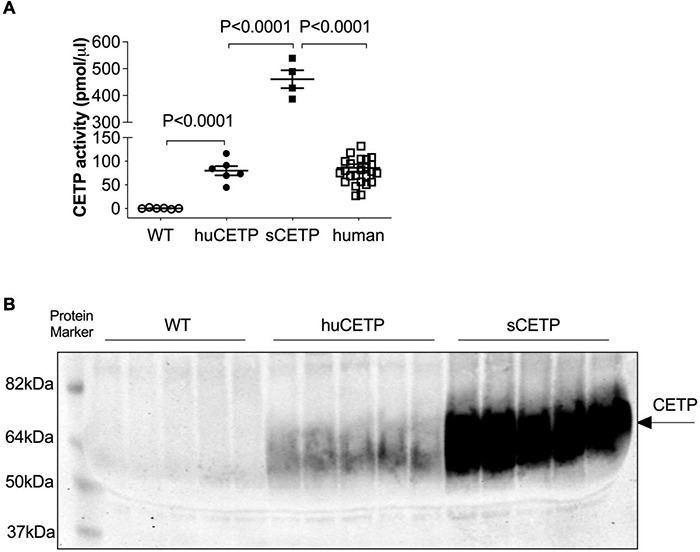
CETP expression in huCETP mice is comparable to that in humans. **(A)** Blood CETP activity from chow-fed WT (*n* = 6), huCETP (*n* = 6), and simian CETP mice (*n* = 4) and humans (*n* = 26). Statistical analysis by one-way ANOVA. **(B)** Blood was collected from age-matched female WT, huCETP and simian CETP (sCETP) mice. Two microliters of serum were used for detecting CETP protein levels in blood with immunoblotting. Protein markers were indicated on the left and CETP specific bands were indicated by an arrow on the right. Non-specific bands or background was not denoted.

Mice carrying the human CETP minigene (huCETP) have a tissue expression pattern of CETP that is similar to humans (Jiang et al., 1992). To better understand the roles of CETP in lipid metabolism relevant to humans, we used huCETP mice that have CETP expression in the tissues similar to humans (Supplementary Figure 1) compared with CETP expression in the FANTOM5 dataset (Uhlen et al., 2015). We measured blood CETP activity in huCETP transgenic mice and their wild-type (WT) littermates that naturally lack CETP expression, simian CETP (sCETP) transgenic mice, and humans. Serum CETP activity in huCETP mice was higher in huCETP mice than their WT littermates and comparable to blood CETP activity in humans but lower than sCETP mice (Figure 1A). In addition, we compared serum CETP protein amounts in huCETP, WT littermates, and sCETP mice. CETP protein was not detectable in WT mice, and serum CETP protein levels were higher in mice overexpressing the simian CETP than huCETP mice (Figure 1B). Thus, huCETP mice display a tissue expression of CETP and blood activity levels similar to humans.

### Expression of the Human Cholesteryl Ester Transfer Protein Minigene Protects Against Diet-Induced Insulin Resistance in Female Mice

Mice expressing the human CETP minigene and their WT littermates were fed a high-fat diet (HFD) for 6 months. The preliminary data showed that there were no significant differences between male huCETP and WT male littermates in glucose infusion rate during euglycemic-hyperinsulinemic clamps (Supplementary Figure 1B). In addition, based on the previous report that overexpression of simian CETP protected against insulin resistance only in female mice (Cappel et al., 2013), we further investigate the metabolic impact of human CETP minigene in female mice in the current study.

**FIGURE 2:**
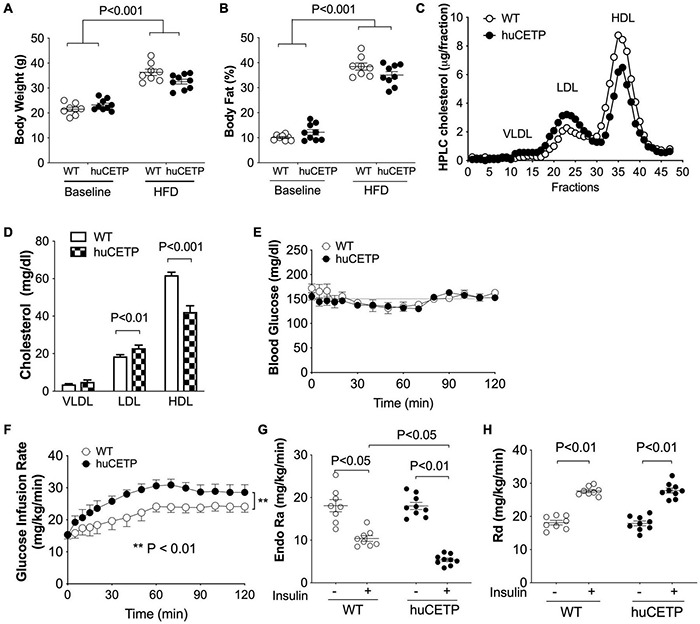
huCETP mice are protected from diet-induced insulin resistance. Female huCETP mice and WT littermates were fed a high-fat diet for 6 months (*n* = 8–9). **(A)** Body weight before and after high-fat diet (HFD) feeding. **(B)** Body composition before and after HFD feeding. **(C)** Serum lipoproteins were separated by HPLC and lipoprotein cholesterol was determined as described in section “Materials and Methods.” **(D)** Bar graph of lipoprotein cholesterol levels. Significant differences were determined using the Student’s *t*-test. **(E)** Euglycemia was maintained at ∼150 mg/dL during the insulin clamp. **(F)** Glucose infusion rate (GIR) to maintain euglycemia was higher in huCETP mice than in their WT littermates. **(G)** Insulin suppression of hepatic glucose production (Endo Ra) was more pronounced in huCETP mice than in their WT littermates. **(H)** Insulin-stimulated glucose disappearance rate (Rd) was similar between groups. Data shown are mean ± SEM. Significant differences were determined by repeated measures by both factors 2-way ANOVA with Bonferroni’s multiple comparison test except panel **(D)**.

HFD-feeding increased body weight and adiposity in both female huCETP and WT female littermates, and changes in body weight were not significantly affected by huCETP expression (Figures 2A,B). There were no significant differences in fasting blood glucose, insulin, TG, or cholesterol concentrations between huCETP and WT female mice (Table 1). HDL-cholesterol levels were decreased by 38% and LDL-cholesterol levels were increased by 23% while VLDL-cholesterol levels were not significantly changed in huCETP mice compared to their WT littermates (Figures 2C,D). We performed a euglycemic-hyperinsulinemic clamp study to determine insulin sensitivity for huCETP mice. The glucose infusion rate (GIR) required to maintain euglycemia under hyperinsulinemic conditions is an index of insulin sensitivity. The GIR was significantly higher in female huCETP than WT female littermates (Figures 2E,F), suggesting insulin sensitivity is higher for huCETP mice than WT littermates in females. Hepatic glucose production (endogenous rate of appearance, EndoRa, an index of liver insulin sensitivity) was suppressed by insulin in WT littermates and was further suppressed in huCETP mice during the insulin clamp (Figure 2G). Glucose disposal (rate of disappearance, Rd, an index of muscle insulin sensitivity) was not affected by CETP expression in huCETP mice in comparison to their WT littermates (Figure 2H).

**TABLE 1:** Blood chemistry during fasting and clamps.

	Wild-type littermates	huCETP
**Fasting**		
Glucose (mg/dl)	172.2 ± 8.9	155.6 ± 8.7
Insulin (pg/ml)	2,377 ± 415	1,726 ± 341
TG (mg/dl)	63.9 ± 6.9	64.5 ± 2.0
Cholesterol (mg/dl)	118.6 ± 6.3	106.8 ± 8.9
**Clamps**		
Insulin (pg/ml)	2,530 ± 478	2,164 ± 301
IL-6 (pg/ml)	407 ± 45	333 ± 62
Resistin (pg/ml)	3,906 ± 396	5,001 ± 597
MCP-1 (pg/ml)	156.4 ± 12.4	109.4 ± 14.1
Leptin (pg/ml)	2,563 ± 366	2,147 ± 380

*Data shown are mean ± SEM, n = 8–9. Statistical analysis was performed by student t-test. TG, triglycerides; IL-6, interleukin-6; MCP-1, monocyte chemoattractant protein-1.*

**FIGURE 3:**
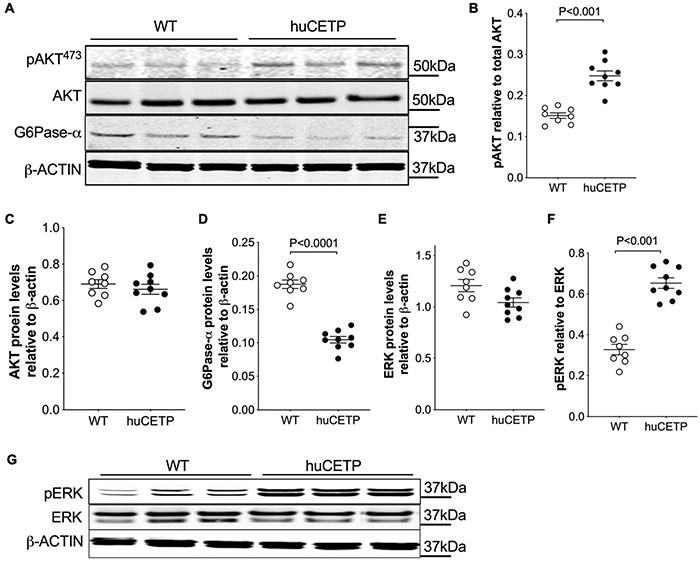
Insulin-induced phosphorylation of liver AKT and ERK and suppression of liver G6Pase-α are more pronounced in female huCETP mice than in their WT littermates. **(A–C)** Insulin-mediated phosphorylation of liver AKT(^473^ser) was increased in huCETP mice shown by immunoblotting **(A)** and its quantification **(B,C)**. **(A,D)** Immunoblotting showed decreased liver G6Pase-α protein levels in huCETP mice. **(E–G)** Increased phosphorylation of liver ERK in huCETP mice. Liver tissue was collected after insulin infusion at the end of the insulin clamp study. Protein markers were indicated on the right side of the blots and β-ACTIN was used as the loading control. The Student’s *t*-test was used for statistical analysis, *n* = 8–9.

The phosphorylation of AKT at ^473^Ser in insulin signaling in the liver was modestly increased in huCETP mice in comparison to their WT littermates (Figures 3A–C). G6Pase-α expression in the liver was decreased by 45% (Figures 3A,D), consistent with the lower EndoRa in huCETP mice. Phosphorylation of ERK (pERK) in the liver was increased by over two-fold in huCETP mice compared to WT littermates (Figures 3E–G), consistent with the previous report that pERK was increased in the liver of sCETP mice (Cappel et al., 2013). These results demonstrate an enhanced activity with regard to insulin signaling pathways in the liver of huCETP mice.

**FIGURE 4:**
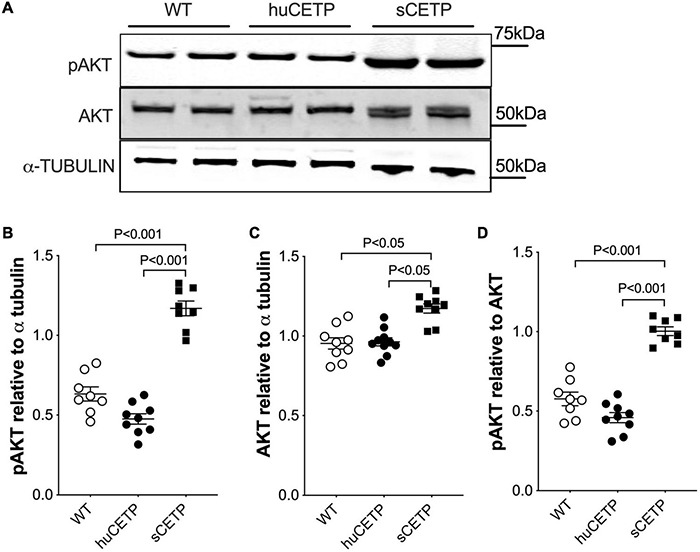
AKT and AKT phosphorylation in skeletal muscles. **(A)** Immunoblots for AKT and pAKT in vastus medialis muscle from HFD-fed wild type, huCETP, and sCETP mice. Quantification of pAKT, AKT, and pAKT/AKT ratio was shown in **(B–D)**. α-Tubulin was used as the loading control for muscle tissues and protein markers were indicated on the right side of the blots. One-way ANOVA was used for statistical analysis, *n* = 8–9.

We previously reported that mice over-expressing simian CETP had enhanced glucose disposal (Rd) compared to WT littermates (Cappel et al., 2013), which was not seen in the current study with huCETP transgenic mice. To address this question, we investigated the insulin signaling pathway in vastus medialis muscle in WT, huCETP, and sCETP mice. As shown in Figure 4, AKT and its phosphorylation were increased in sCETP mice but not in huCETP mice. This may either relate to the several-fold increase in CETP activity in sCETP vs. huCETP mice, or the differences in promoter activity of the sCETP mice which is driven by the mouse metallothionein (MT1) promoter vs. the natural flanking sequences for the huCETP mice. These results together suggest that, unlike sCETP mice, the liver mediates most of the beneficial effects of CETP on insulin sensitivity in huCETP mice.

### Transgenic Expression of Cholesteryl Ester Transfer Protein Improves Fatty Liver in Human Cholesteryl Ester Transfer Protein Minigene Mice

**FIGURE 5:**
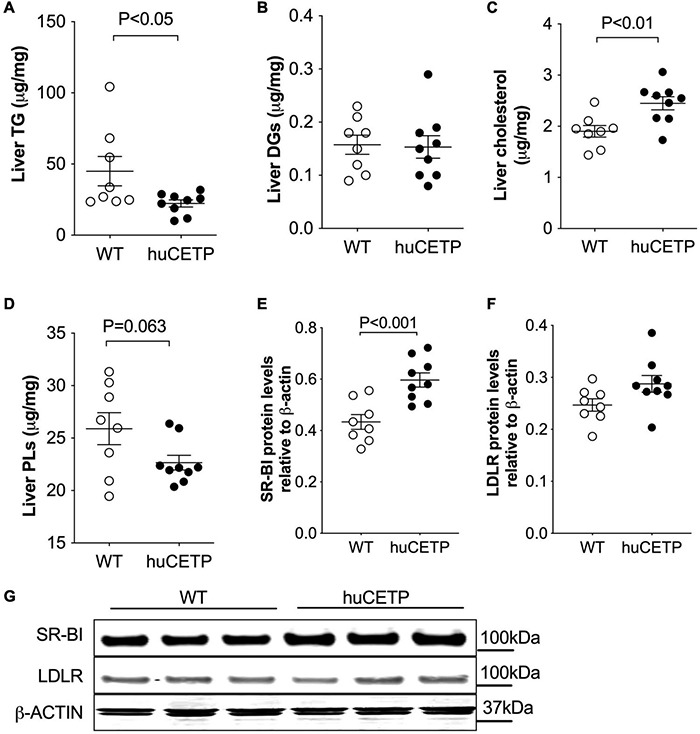
Changes in liver lipid content and expression of liver lipoprotein receptors. **(A–D)** Lipid content for triglycerides (TGs), diacylglycerol (DGs), cholesterol, and phospholipids (PLs) in the liver are shown in **(A–D)**, respectively. **(E–G)** Immunoblots for SR-BI and LDLR proteins in the liver were shown in **(G)**. Protein markers were indicated on the right side of the blots and β-ACTIN was used as the loading control. Quantifications of the blots for SR-BI and LDLR were shown in **(E,F)**. The Student’s *t*-test was used for statistical analysis, *n* = 8–9.

Fatty liver is a known contributor to insulin resistance (Kitade et al., 2017). Thus, we compared liver lipid content in huCETP and WT littermate mice. Liver TG content was 50% lower in huCETP mice than in their WT littermates, while liver diacylglycerol (DG) levels were similar between huCETP and WT mice (Figures 5A,B and Supplementary Figure 3A). Liver cholesterol content was higher in huCETP mice than in their WT littermates (Figure 5C), consistent with known effects of CETP to promote the delivery of cholesterol to the liver and to decrease serum HDL-cholesterol levels (Figures 2C,D). Liver phospholipid levels (PLs) trended lower but were not statistically different between groups (Figure 5D).

To understand whether the huCETP expression would modify pathways that promote liver lipid uptake, we investigated liver SR-BI and LDLR protein levels. The expression of huCETP increased liver SR-BI protein levels and did not affect liver LDLR protein levels (Figures 5E–G). These results indicate a correlation between the decreased HDL-cholesterol level and increased liver cholesterol uptake and liver cholesterol content in huCETP mice.

### Fatty Acid β-Oxidation Pathways Are Upregulated in the Liver of Human Cholesteryl Ester Transfer Protein Minigene Transgenic Mice

**FIGURE 6:**
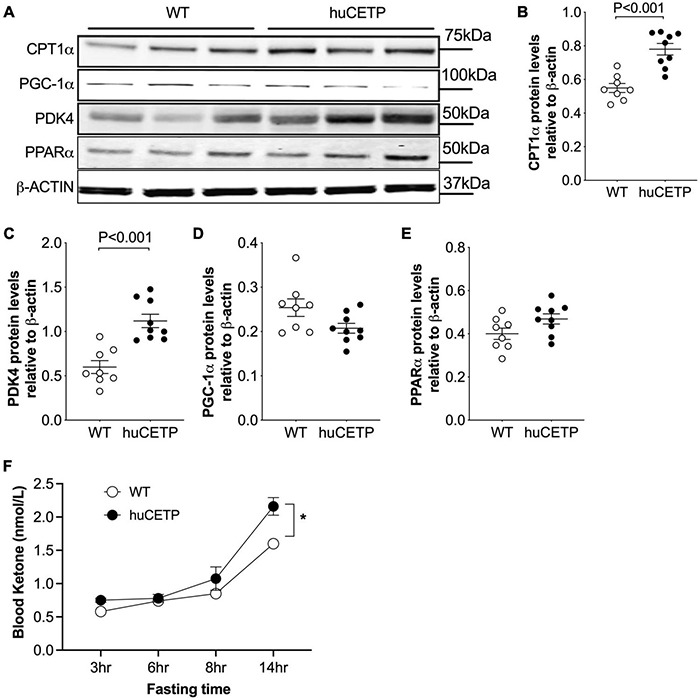
Liver fatty acid β-oxidation are increased *in vivo* with huCETP mice. **(A)** Western blots of liver CPT1, PGC-1α, PDK4, and PPARα from huCETP and WT mice. β-ACTIN was used as the loading control and protein markers were indicated on the right side of the blots. Quantification of western blots for each protein shown in **(B–E)** Data shown in B-E are mean ± SEM, *n* = 8–9. Significant differences were determined by the Student’s *t*-test. **(F)** Fasting blood ketone body levels in huCETP and WT littermates. *n* = 5, *P < 0.05 determined by repeated measured by both factors 2-way ANOVA.

To understand the mechanisms for the decreased liver TG content in huCETP mice, we investigated pathways for liver lipid oxidation and synthesis. Carnitine palmitoyltransferase 1α (CPT1α) transports fatty acids into mitochondria for β-oxidation in the liver (Postic and Girard, 2008). Liver CPT1α protein amounts were increased in huCETP mouse liver (Figures 6A,B). The upregulation of pyruvate dehydrogenase kinase 4 (PDK4) is closely associated with increased fatty acid oxidation (Pettersen et al., 2019). Liver protein levels of PDK4 were also increased in huCETP transgenic mice (Figures 6A,C). Both *Cpt1α* and *Pdk4* are target genes of PPARα-activated transcription. However, the liver PPARα protein amounts were not altered by the transgenic expression of huCETP, neither were the PGC-1α protein amounts (Figures 6A,D,E). We next measured fasting blood ketone levels, an index of whole-body fatty acid oxidation. As shown in Figure 6F, blood ketone levels gradually increased during the fasting period in both genotypes of mice. The overall blood ketone levels were higher in huCETP mice than their WT littermates during a 14-h fasting period (Figure 6F). These data suggest that fatty acid oxidation rate in the liver was upregulated in huCETP female mice.

With regard to TG synthesis pathways, liver acetyl-CoA carboxylase (ACC) and its phosphorylated form pACC were not significantly altered by the expression of human CETP minigene (Supplementary Figures 3B–E). Diacylglycerol O-acyltransferase 1 and 2 (DGAT1/2) protein amounts in the liver were similar between huCETP and WT mice (Supplementary Figures 3B,F,G). These results suggest that reduced liver TG content in huCETP mice may be due to the increased hepatic fatty acid β-oxidation rather than decreased TG synthesis.

### Liver Long-Chain Fatty Acid Levels Are Increased in Human Cholesteryl Ester Transfer Protein Minigene Transgenic Mice

**FIGURE 7:**
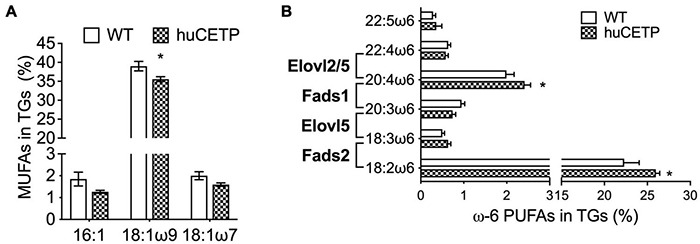
Liver fatty acid composition and protein levels of enzymes that regulate long-chain PUFA biosynthesis pathways. The liver fatty acid composition was determined by the Vanderbilt Medical Center Lipid Core. 16C- and 18C-monounsaturated fatty acids in TGs are shown in **(A)**. Long-chain ω6 polyunsaturated fatty acids in TGs and the catalytic enzyme for each step are shown in **(B)**. Significant differences were determined by the Student’s *t*-test, *n* = 8–9, *P < 0.05.

To understand the mechanisms that drive hepatic fatty acid β-oxidation in huCETP transgenic mice, we further explored the ligand-activators for PPARα signaling since both liver PPARα and PGC-1α protein levels were not changed. Long-chain polyunsaturated fatty acids (LC-PUFAs) are important ligands to activate PPARα (Contreras et al., 2013). Thus, we measured the fatty acid composition in the liver of huCETP mice and WT littermates. The monounsaturated fatty acid 18:1ω9 was 11% lower in huCETP mouse liver than WT mouse liver (Figure 7A and Supplementary Figure 4A). For LC-PUFAs, linoleic acid (18:2ω6) and arachidonic acid (20:4ω6) were higher in the huCETP mouse liver than WT mouse liver (Figure 7B and Supplementary Figure 4A). Arachidonic acids and their polyunsaturated fatty acid analogs are known to bind to PPARα and activate gene transcription (Contreras et al., 2013). Serum inflammatory factors were not significantly different between huCETP and WT littermates (Table 1). Fatty acid species in liver phospholipids were not significantly different between huCETP and WT littermates (Supplementary Figure 4B).

**FIGURE 8:**
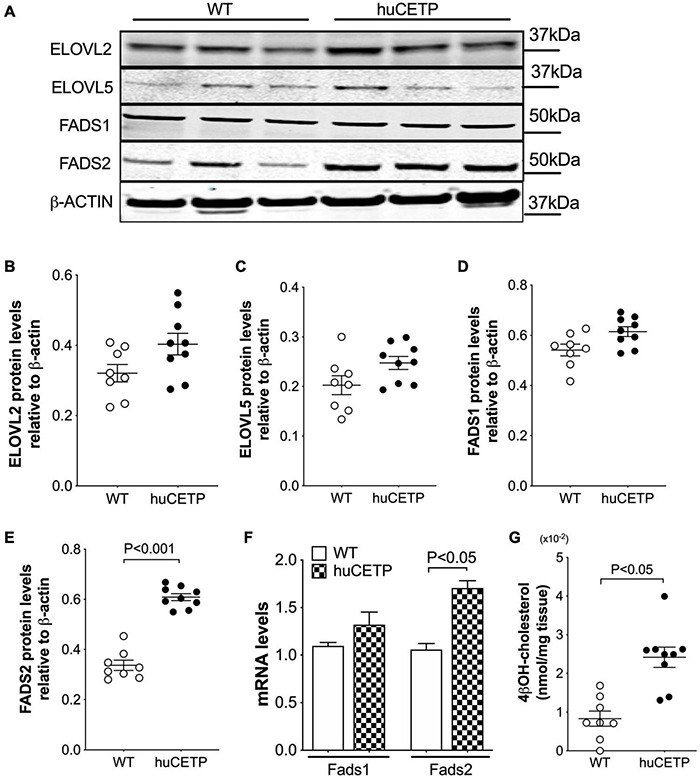
Liver protein levels of enzymes that regulate long-chain PUFA biosynthesis pathways. Immunoblots for enzymes in the long-chain polyunsaturated fatty acid biosynthesis **(A)**. Quantification of SCD1, ELOVL2, ELOVL5, FADS1, and FADS2 is shown in **(B–F)**, respectively. β-ACTIN was used as the loading control and protein markers were indicated on the right side of the blots. **(F)** Liver mRNA levels of *Fads1* and *Fads2* were determined with qPCR, 18S RNA was used as the internal control. **(G)** Liver 4βOH-cholesterol levels were increased in huCETP mice. Significant differences were determined by repeated measures by both factors 2-way ANOVA with Bonferroni’s multiple comparison test for panel G and the Student’s *t*-test where applies, *n* = 8–9.

We then examined liver protein levels of enzymes that regulate elongation of very-long-chain fatty acids (ELOVL) and fatty acid desaturases (FADS) in LC-PUFA biosynthesis pathways (Figure 8 and Supplementary Figure 4C). ELOVL2/5 and stearoyl-CoA desaturase 1 (SCD1) protein levels in the liver were not significantly different between huCETP mice and WT littermates (Figures 8A–C and Supplementary Figure 3). Gene expression of fatty acid desaturases (*Fads1/2*) was generally higher in huCETP mouse liver, with a 165% higher level of *Fads2* mRNA than WT littermates (Figure 8F). Protein levels of FADS1 did not change, while protein levels of FADS2 were increased in the liver in huCETP mice (Figures 8A,D,E). These data suggest that the biosynthesis pathways for long-chain unsaturated fatty acids were upregulated in the liver of huCETP transgenic mice and long-chain unsaturated fatty acids further promoted β-oxidation as ligand-activators for PPARα.

**FIGURE 9:**
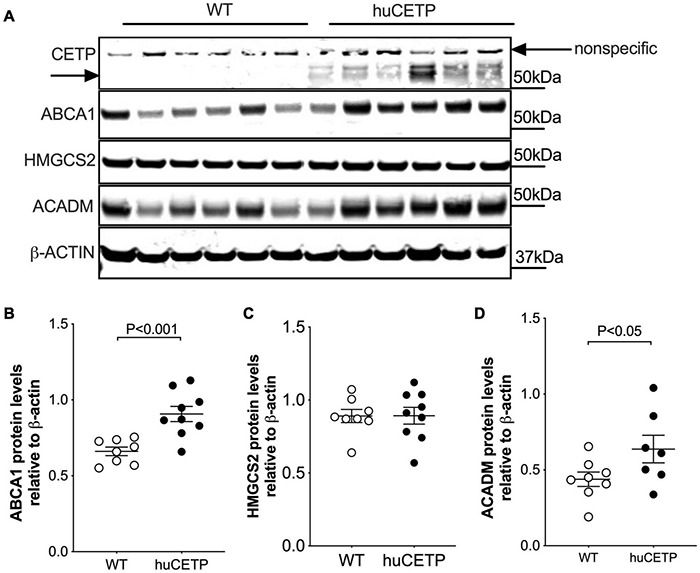
Liver ABCA1 and ACADM protein amounts are increased in huCETP mice. **(A)** Immunoblots for protein expression of liver CETP, ABCA1, HMGCS2, and ACADM in huCETP and wild type littermates. β-ACTIN was used as the loading control and protein markers were indicated on the right side of the blots. Quantification of western blots for each protein shown in **(B–D)** Data shown are mean ± SEM, *n* = 8–9. Significant differences were determined by the Student’s *t*-test.

CETP is known to increase the delivery of cholesteryl esters to the liver. We also examined levels of oxysterols which are intermediates in the conversion of cholesteryl esters to bile acids and ligands for Liver X receptor (LXR) α and β. We found that the transgenic expression of human CETP minigene increased levels of 4βOH-cholesterol in the liver but did not lead to a significant change in other measured oxysterol levels (Figure 8H and Supplementary Figure 5). We were not able to successfully blot for LXR α or β protein levels from the liver with commercially available antibodies. Furthermore, liver protein amounts of ATP-binding cassette transporter A1 (ABCA1), a target gene of LXR, were increased in huCETP mice compared to WT littermates (Figures 9A,B). Liver hydroxymethylglutaryl-CoA synthase 2 (HMGCS2) protein levels were not significantly different between huCETP and WT mice (Figure 9C), but liver medium-chain specific acyl-CoA dehydrogenase (ACADM) protein amounts were modestly higher in huCETP mice than WT littermates (Figures 9A,D). While both *Hmgcs2* and *Acadm* are PPARα target genes, HMGCS2 is a regulatory enzyme for ketogenesis and ACADM is a regulatory enzyme for fatty acid oxidation (Stenson et al., 2009). Our results indicate that the increased fasting ketone body levels in huCETP mice were largely due to the upregulation of enzymes in liver fatty acid oxidation pathways rather than the regulation of enzymes in ketogenesis.

## Discussion

In this study, we show that female transgenic mice expressing the human CETP minigene containing its natural regulatory elements are protected from diet-induced insulin resistance. With tracers, we show that this improved insulin sensitivity was attributed to the improved insulin-mediated suppression of hepatic glucose production (EndoRa) during the euglycemic-hyperinsulinemic clamp. Improved liver insulin signaling pathways were also associated with decreased liver TG content in huCETP transgenic mice. Furthermore, expression of the human CETP minigene increased liver LC-PUFA levels and stimulated hepatic fatty acid β-oxidation pathways, which is likely responsible for the limited liver TG content and improved whole-body insulin sensitivity during high-fat diet feeding.

Expression of the human CETP minigene limited diet-induced fatty liver by promoting liver fatty acid β-oxidation in female mice. The upregulation of fatty acid β-oxidation signaling in the liver has been reported in mice overexpressing the simian CETP gene (Cappel et al., 2013; Palmisano et al., 2016). In simian CETP transgenic mice, the upregulation of *Cpt1a* and *Cpt2* gene expression was accompanied by elevated mRNAs of *Pparα*, *Acox*, and *Acadm*, genes that govern fatty acid oxidation in the liver (Palmisano et al., 2016). In the present study, the increased protein amounts of CPT1α and PDK4 were associated with increased fasting blood ketone body levels in huCETP mice (Figure 6F). Reciprocally, pharmaceutical inhibition of CETP activity in circulation by anacetrapib leads to decreased CPT1α protein amounts and hepatic fatty acid β-oxidation in the liver of simian CETP mice (Zhu et al., 2018a). These studies suggest that CETP has a role in limiting fatty liver and improving liver insulin sensitivity with obesity. The effects of huCETP on insulin sensitivity appear to be sex-specific in female mice, which was also shown with simian CETP over-expression (Cappel et al., 2013). Studies have shown that the association between CETP SNPs and CVD risk factors, such as LDL- and HDL-cholesterol levels, is sex-specific (Agerholm-Larsen et al., 2000; de Vries et al., 2005; Anagnostopoulou et al., 2009). With a small sample size (*n* = 10/group), we did not observe sex differences in blood CETP activity but observed a reverse association between plasma TG levels and CETP activities in men only (Supplementary Figure 2).

With the huCETP mouse model, we demonstrate that CETP expression increased hepatic fatty acid β-oxidation, which is likely mediated by PPARα pathway. It has been shown that the activation of PPARα improves steatosis, inflammation, and fibrosis in rodent models of non-alcoholic fatty liver by promoting peroxisomal and mitochondrial β-oxidation (Hashimoto et al., 2000; Xu et al., 2002; Staels et al., 2013). Unlike simian CETP transgenic mice where PPARα expression was upregulated at the mRNA levels, expression of the human CETP minigene activated PPARα at posttranslational levels by the increased LC-PUFA levels in the liver. FADS2 is the rate-limiting enzyme for the formation of endogenous LC-PUFAs and both its mRNA and protein levels have been up-regulated in huCETP mice. The increased linoleic acid levels in the huCETP mouse liver are likely from the diet as an essential fatty acid, which might be associated with highly expressed CETP in the small intestine in huCETP mice (Jiang et al., 1992). The increased linoleic acid levels, as substrates, may partially contribute to the increased LC-PUFAs in the huCETP mouse liver for PPARα activation. FADS2 is a target gene of LXR (Varin et al., 2015; Zhang et al., 2016). FADS and LC-PUFA biosynthesis are upregulated by LXR activation in mouse macrophages, and LXR activation promotes long-chain fatty acid oxidation as well as PDK4 and ABCA1 protein levels in human cells (Stenson et al., 2009; Varin et al., 2015). The activation of LXR in the huCETP mouse liver was associated with increased cholesterol delivery to the liver by CETP and increased cholesterol metabolites including 4βOH-cholesterol (Figure 8G). 4βOH-cholesterol is a ligand-activator for LXR transcriptional activity (Salonurmi et al., 2020). Of note, LXR activation was not associated with increased lipogenesis that could be mediated by sterol regulatory element-binding protein (SREBP). In huCETP mice where sterol delivery to the liver is increased, nuclear translocation of SREBP would likely be suppressed due to the inactivation of the cleavage-activating protein (SCAP) (Wang et al., 1994; Janowski et al., 1996). CETP is a target gene of LXR and can be up-regulated by dietary cholesterol in humans (Jiang et al., 1992; Tato et al., 1995; Luo and Tall, 2000; de Vries et al., 2005). With dietary cholesterol, expressing of CETP promotes cholesterol reverse transport and decreases atherosclerosis in SR-BI null mice (Harder et al., 2007), but severely aggravates atherosclerosis in apoE3-leiden mice (Westerterp et al., 2006). The impact of CETP regulation by dietary cholesterol on CVD risks including dyslipidemia and insulin resistance is not yet conclusive. Interestingly, we for the first time show that CETP expression limited diet-induced liver fat via LXR, indicating the physiological significance of CETP in mediating lipid metabolism in humans when its expression is induced by diet. Furthermore, pharmaceutical inhibition of CETP activity in the blood suppressed fatty acid oxidation in the liver (Clark et al., 2006). Altogether suggest that the decreased HDL-cholesterol levels mediated by blood CETP increased liver cholesterol uptake and metabolism, which may subsequently modify liver lipid metabolism and whole-body insulin sensitivity.

The huCETP mouse model in the current study along with previous studies using sCETP mice seems to show that the magnitude of improved insulin signaling relates to the magnitude of increased CETP activity. The improved whole-body insulin sensitivity driven by the suppression of liver G6pase-α and hepatic glucose production was seen in both huCETP and simian CETP mice (Cappel et al., 2013). Though not directly comparable, qualitatively, the improvement in insulin sensitivity, i.e., the average glucose infusion rate (230% in simian CETP in our prior study (Cappel et al., 2013) vs. 130% in huCETP mice in this study), seems to be associated with the suppression of G6pase-α (86% in simian CETP in our prior study (Cappel et al., 2013) vs. 45% in huCETP mice in this study) and blood CETP activity (460 pmol/μL in simian CETP vs. 80 pmol/μL huCETP mice, both shown in Figure 1). The over-expression of CETP was associated with the upregulation of liver PPARα at the transcriptional levels in sCETP mice while the mild expression of CETP was associated with the upregulation of PPARα at the posttranslational levels in huCETP mice. Furthermore, the increased insulin sensitivity associated with the increased *Cpt1b* mRNAs in skeletal muscles was only seen when CETP was abundantly expressed in sCETP mice (Cappel et al., 2013). Together these studies indicate that the protective roles of CETP with regard to lipid metabolism and insulin action depend on the CETP activities.

In a previous study by Raposo and colleagues, blood glucose levels were not significantly different between huCETP and WT mice during either glucose- or insulin-tolerance test (Raposo et al., 2016). This difference may be due to 1) the modest expression of huCETP is not sufficient to see its metabolic effects after a shorter-term HFD feeding in their study; and/or 2) the sensitive nature of the insulin clamp techniques used to define insulin sensitivity in our study, particularly with regard to liver insulin sensitivity which is the primary organ impacted. Our results for the improvement of insulin sensitivity during a chronic HFD feeding demonstrate the significance of using the huCETP mouse model, which likely has more physiologically relevant levels of CETP to study the physiological roles of CETP. Raposo et al. (2021) recently demonstrated that CETP expression attenuates adiposity in both huCETP and sCETP transgenic mice by enhancing lipolysis and energy expenditure. In clinic, blood CETP mass and activity are increased with obesity in non-diabetic humans (Arai et al., 1994; Magkos et al., 2009) and the reduced expression of CETP is associated with the development of type II diabetes in obese subjects (Kahri et al., 1994; MacLean et al., 2000, 2005). These reports indicate the protective roles of CETP for diabetic insulin resistance, and the results of our studies shed a light on the underlying mechanisms for these protective roles of CETP.

The transgenic expression of human CETP in mice limits liver lipid accumulation and improves insulin resistance during high-fat diet feeding in females. We demonstrate increased fatty acid β-oxidation in the liver of huCETP mice, which was associated with increased levels of LC-PUFA and the activation of PPARα-target genes in fatty acid β-oxidation signaling pathways. CETP is well-known for its role in modifying lipid distribution between lipoproteins in circulation, which is correlated with the risks for cardiovascular disease. However, the physiological consequences of the CETP-modified changes in lipoprotein lipids in other tissues such as the liver are yet not clear. We have studied CETP biology in models that parallel the eightfold range of CETP activity seen in humans (Tato et al., 1995; de Vries et al., 2005), from over-expression of the simian CETP gene to levels similar to humans with the huCETP model. It may be informative that even at the lower levels of CETP seen in humans that CETP modifies hepatocyte function with regard to lipid metabolism and liver insulin signaling pathways. Our findings for the protective roles of CETP for diet-induced fatty liver and insulin resistance specific in females may provide insight to explain why most clinical trials with CETP inhibitors failed in improving the risks for cardiovascular disease. Furthermore, our findings support a growing picture of the physiological importance of CETP in the adaptation to chronic over-nutrition. Considering the sex-specific findings in both simian and huCETP in female mice, CETP might contribute to sex-specific adaptation to nutrient excess beneficially in females.

## Data Availability Statement

The original contributions presented in the study are included in the article/Supplementary Material, further inquiries can be directed to the corresponding author/s.

## Ethics Statement

The animal study was reviewed and approved by the Institutional Animal Care and Use Committee at Vanderbilt University.

## Author Contributions

LZ wrote the manuscript and researched data. JA, SC, TL, YDP, BL, KF, CF, and H-YK researched data and reviewed the manuscript. ML and JS researched data and contributed to discussion and reviewed and edited manuscript. All authors contributed to the article and approved the submitted version.

## Conflict of Interest

The authors declare that the research was conducted in the absence of any commercial or financial relationships that could be construed as a potential conflict of interest.

## Publisher’s Note

All claims expressed in this article are solely those of the authors and do not necessarily represent those of their affiliated organizations, or those of the publisher, the editors and the reviewers. Any product that may be evaluated in this article, or claim that may be made by its manufacturer, is not guaranteed or endorsed by the publisher.

## Abbreviations

AA: arachidonic acid
ACC: acetyl-CoA carboxylase
ACADM: medium-chain specific acyl-CoA dehydrogenase
ApoA1: apolipoprotein A1
CETP: cholesteryl ester transfer protein
CPT1: carnitine palmitoryltransferase 1
CVD: cardiovascular disease
DGAT: diacylglycerol O-acyltransferase
ERK: extracellular signal-regulated kinase
ELOVL: elongation of very long chain fatty acid
FADS1: Δ 5 fatty acid desaturase
FADS2: Δ 6 fatty acid desaturase
FAO: Fatty acid oxidation
HDL: high-density lipoprotein
HFD: high-fat diet
HGP: hepatic glucose production
HMGCS2: hydroxymethylglutaryl-CoA synthase 2
HPLC: high-performance liquid chromatography
IL-6: interleukin-6
LC-PUFA: long-chain polyunsaturated fatty acid
LDL: low-density lipoprotein
LDLR: low-density lipoprotein receptor
LXR: liver X receptor
MCP-1: monocyte chemoattractant protein-1
PPAR: peroxisome proliferator-activated receptor
PGC-1 α: PPAR gamma coactivator 1 α
PDK4: pyruvate dehydrogenase kinase 4
SCD1: stearoyl-CoA desaturase 1.
