# Supplementary material for: Expressing the Human Cholesteryl Ester Transfer Protein Minigene Improves Diet-Induced Fatty Liver and Insulin Resistance in Female Mice
Source: Front Physiol. 2022 Jan 10;12:799096. doi: 10.3389/fphys.2021.799096 (PMC8784660; doi:10.3389/fphys.2021.799096)
Supplement: Supplementary file 1 [file Data_Sheet_1.PDF]

Supplemental Figure 1

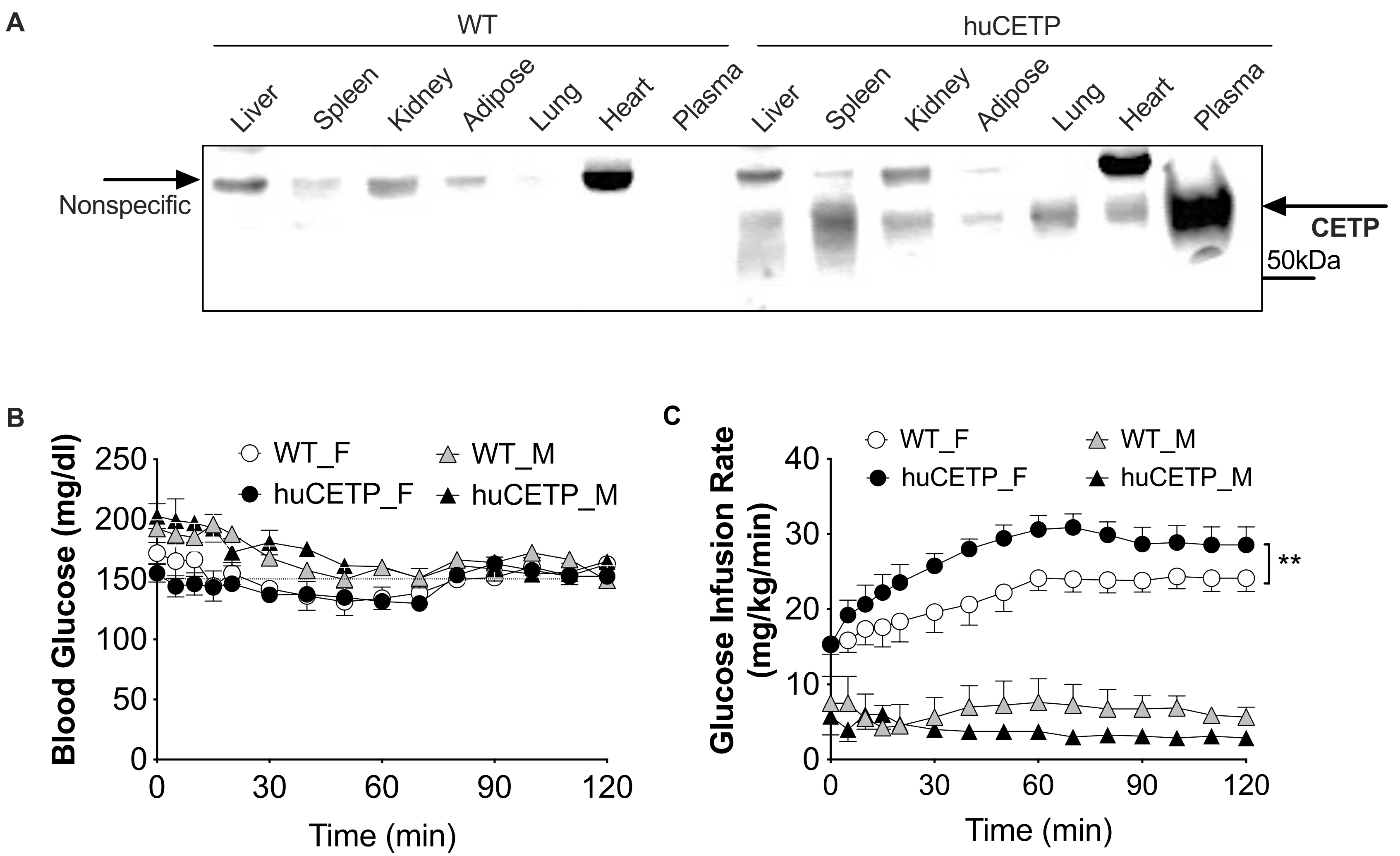

Supplemental Figure 1. Transgenic expression of huCETP protected against diet-induced insulin resistance in female mice not in male mice. A. Western blots of tissue expression of CETP in huCETP mice and their wild type (WT) littermates. **B.** Euglycemia was maintained at ~150 mg/dl during the insulin clamp. **C.** Glucose infusion rate (GIR) to maintain euglycemia was higher in huCETP female mice than in WT female littermates. No significant difference was seen between huCETP male mice and WT male littermates. Data shown are mean±SEM, n=8-9/group for female mice; n=4/group for male mice. Significant differences were determined by repeated measurs by both factors 2-way ANOVA. **P<0.01.

Supplemental Figure 2

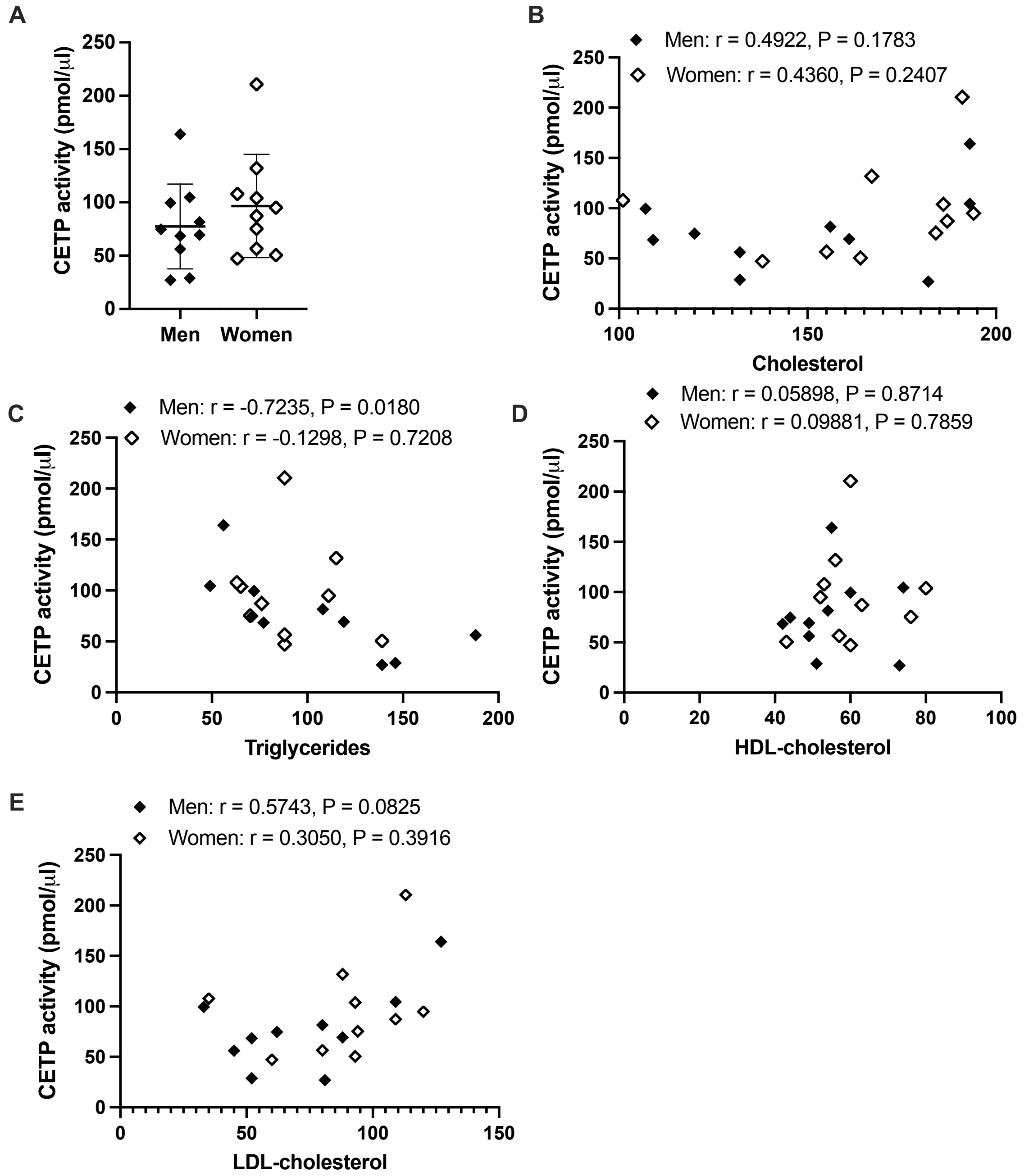

Supplemental Figure 2. Blood CETP activity and its corelation with blood lipids in men and women. A. Blood CETP activity was not significantly different between men and women. n=10/group, Student t-test. **B-E.** Blood CETP activity was not correlated with blood total cholesterol (**B**) or HDL-cholesterol (**D**) in either men or women. Blood CETP activity was negatively related to blood triglyceride levels in men but not in women (**C**). Blood CETP activity was generally correlated with LDL-cholesterol (r=0.4652, P=0.03897) when results from men and women were pooled, but not sigfinicantly correlated with LDL-cholesterol in either men or women (**E**). n=10/group.

Supplemental Figure 3

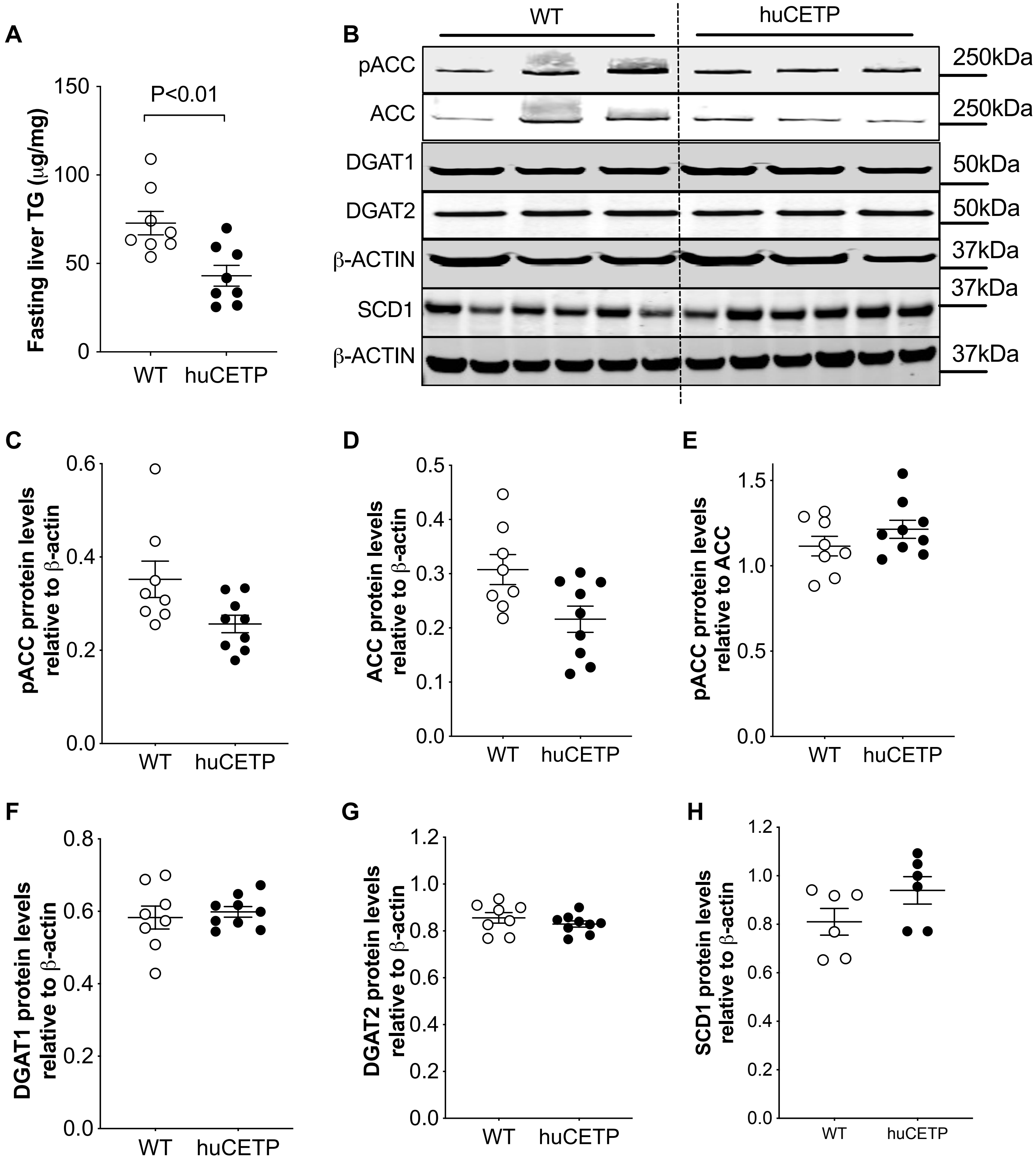

Supplemental Figure 3. Liver lipogenic pathways are not changed by the expression of human *Cetp* minigene. A. Fasting liver TG content from the 3rd cohort female mice (n=8, the Student's *t*-test was used for statistical analysis). **B.** Immunoblots for hepatic lipogenesis enzymes, e.g. ACC and its phosphorylation pACC, DGAT1, DGAT2, and SCD1. β-ACTIN was used as the loading control and protein markers were indicated on the right side of the blots. Quantifications of the blots for pACC (**C**), ACC (**D**), the ratio of pACC to ACC (**E**), DGAT1 (**F**), DGAT2 (**G**), SCD1 (**H**) are shown. The Student's *t*-test was used for statistical analysis, n=8-9.

Supplemental Figure 4

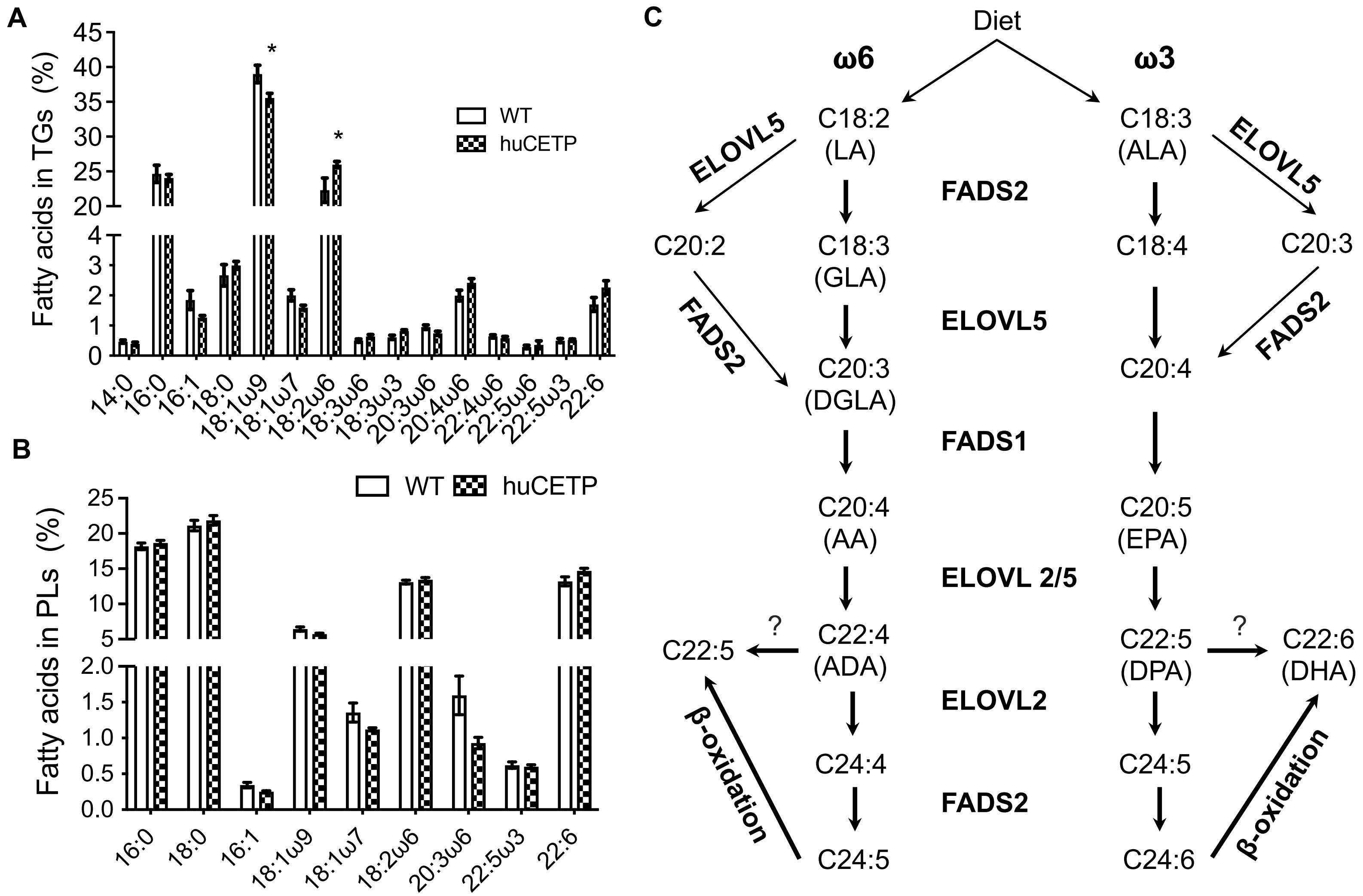

Supplemental Figure 4. Fatty acid composition in triglycerides and phospholipids, and biosynthesis pathways for LC-PUFA. Liver fatty acid composition in triglycerides (**A**) and phospholipids (**B**) were determined by the Vanderbilt Medical Center Lipid Core. Long-chain polyunsaturated fatty acid biosynthesis pathways are shown in (**C**). TG: triglycerides; PL: phospholipid; LA: linoleic acid; GLA: gamma-linolenic acid; DGLA: dihomogammalinolenic acid; AA: arachidonic acid; ADA: adrenic acid; ALA: alpha linoleic acid; EPA: eicosapentanoic acid; DPA: docosapentaenoic acid; DHA: docosahexaenoic acid. ELOVL: elongation of very-long-chain fatty acid; FADS1: 5-fatty acid desaturase; FADS2: 6-fatty acid desaturase. The Student's *t*-test was used for statistical analysis, * $P<0.05$.

Supplemental Figure 5

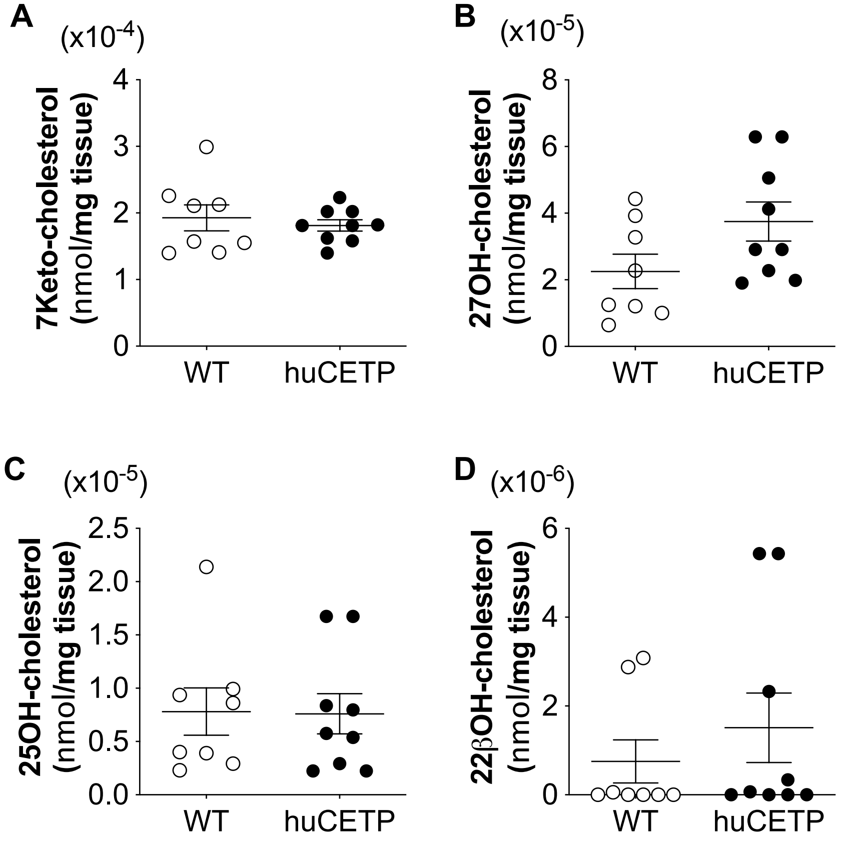

Supplemental Figure 5. Liver cholesterol metabolites. Liver samples were weighed and lysed tissue lysis buffer. The internal standard d_7-ketocholesterol was added and lipids were extracted with Folch solution. Samples were derivatized and oxysterols: 7Keto-cholesterol (A), 27OH-cholesterol (B), 25OH-cholesterol (C), and 22βOH-cholesterol (D) were quantified with MS detections as described in the Methods and Materials section. The Student's *t*-test was used for statistical analysis.
